# Outpatient flexible cystoscopy in urogynaecology: a tertiary hospital’s experience

**DOI:** 10.1007/s11845-025-03891-x

**Published:** 2025-02-01

**Authors:** Parijot Kumar, Saboohi Tariq, Siji Philip, Caroline Hendricken, Fadi Salameh

**Affiliations:** 1 Department of Gynaecology, Rotunda Hospital, Dublin, Ireland; 2 RCSI University of Medicine and Health Sciences, Dublin, Ireland; 3 Connolly Hospital, Dublin, Ireland

**Keywords:** Ambulatory urogynaecology, Flexible cystoscopy, Outpatient, Urogynaecology

## Abstract

**Objective:**

This study is a retrospective evaluation of a new outpatient flexible cystoscopy service in a tertiary care hospital in Dublin, Ireland.

**Design:**

This is a retrospective observational study.

**Setting:**

This study has been held at the Department of Gynaecology, Rotunda Hospital, Dublin.

**Population:**

This included all women who underwent outpatient flexible cystoscopy in Rotunda Hospital between May 2023 to October 2024.

**Materials and methods:**

Retrospective data collection included patient demographics, indications for referrals, cystoscopic findings, post-procedural complications, and cost–benefit analysis.

**Results:**

A total of 77 women underwent flexible cystoscopy. The mean age was 52.5 years ranging from 20 to 85 years. Flexible cystoscopy was successfully completed in all patients, and there were no post procedure complications. The most common indications were as follows: recurrent urinary tract infections (36%) and bladder pain syndrome (36%), followed by overactive bladder symptoms (14%). Fifty-five percent of patients had no abnormal findings. The most common abnormality found was trabeculations (39%), followed by glomerulations (9%). A cost–benefit analysis showed an approximate saving of € 1211 per patient leading to a saving of over € 93,000 over a period of 18 months.

**Conclusion:**

Outpatient cystoscopy is a low-risk, feasible, well tolerated, and safe diagnostic procedure, with no post-procedure complications observed in this study. It avoids the risks of general anaesthesia and is associated with lower procedural costs.

## Introduction

Cystoscopy is an endoscopic examination of the urinary bladder and urethra [1, 2]. It can be done under general or local anaesthetic [1]. Though more commonly used in Urology, cystoscopy also has a significant role in gynaecology, specifically urogynaecology.

The earliest suggestion of a flexible urethrocystoscopy was in 1964, and in 1973, it was first utilised in the field of urology [3, 4]. This diagnostic procedure has been used in the outpatient setting since the 1980s [5]. Common indications include haematuria (frank or microscopic), dysuria, recurrent urinary tract infections (UTIs), bladder pain syndrome (BPS), voiding symptoms, suspected mesh erosions and/or anatomical abnormalities such as vesicovaginal or colovesical fistulae, urethral strictures or diverticulum [1, 6]. The only contraindication to this procedure would be an active/untreated UTI [1].

Cystoscopy can be done with either rigid or flexible cystoscope [1]. Although the technique to perform cystoscopy via rigid or flexible cystoscope is similar, there are a few differences. Flexible cystoscope is narrower and does not require changing of lenses compared to a rigid cystoscope which utilizes a 0- or 30-degree lens and a visual obturator.

The risks associated with this procedure are minimal, including pain and/or bleeding (1 in 10), bladder infection requiring antibiotics (1 in 10 to 1 in 50), temporary catheterisation and/or bladder perforation (less than 1 in 50) [1].

A new service of outpatient flexible cystoscopy was started in the Rotunda Hospital, Dublin, Ireland, in May 2023. The aim of this service was to provide a quick, safe and effective diagnostic option for women waiting for cystoscopy under GA. A referral pathway was devised and was made part of general referral pathway (Appendix 1). Presently, the service only accepts referrals from within the hospital.

The purpose of this study was to evaluate the performance and outcomes of this new service. By analysing patient demographics, indications, procedural findings and complication rates, we sought to assess the safety, feasibility and effectiveness of outpatient flexible cystoscopy. These findings are intended to guide future service planning and quality improvement efforts.

## Methods

### Protocol

A retrospective study was planned, and approval was sought from local clinical audit department to review the charts retrospectively.

### Eligibility criteria

All women who underwent outpatient flexible cystoscopy in Rotunda Hospital between May 2023 to October 2024 were included in the study, and their charts were reviewed retrospectively.

### Data collection process

All charts were retrospectively reviewed through electronic records using Maternal and Newborn Clinical Management System (MN-CMS). Patient age, indication for cystoscopy, cystoscopic findings and any post-procedural complications were recorded. Patients received a phone call from Urogynaecology nurses for follow up on the day of procedure, and charts were reviewed 6 weeks post-procedure to identify any presentations to the hospital with complications.

## Results

A total of 77 women underwent flexible cystoscopy between May 2023 to October 2024. The mean age was 52.5 years ranging from 20 to 85 years. Flexible cystoscopy was successfully completed in all patients, and no procedures were abandoned due to patient discomfort.

It was noted that the most common indications for patient referral were as follows: recurrent UTIs (36%) and BPS (36%), followed by overactive bladder (OAB) symptoms (14%). Table 1 summarises the indications for referral.

**Table 1:** Indications for referral

Indications	Number of patients
Recurrent urinary tract infections (UTIs)	28 (36%)
Bladder pain syndrome (BPS)	28 (36%)
Overactive bladder (OAB)	14 (18%)
Urinary incontinence	9 (12%)
Haematuria	3 (4%)
Suspected mesh erosion	3 (4%)
Voiding dysfunction	1 (1.3%)
Colovesical fistulae	1 (1.3%)

All patients (100%) had normal urothelium with no cancerous pathology. Most common abnormality found was trabeculations (39%), followed by glomerulations (9%). The cystoscopy findings are summarised in Table 2.

**Table 2:** Cystoscopy findings

Cystoscopy findings	Number of patients
Normal urothelium	77 (100%)
Trabeculations	30 (39%)
Glomerulations	7 (9%)
Hyperaemia	6 (8%)
Cystitis cystica	2 (3%)
Incomplete filling	1 (1.3%)
Debris	1 (1.3%)
Mesh erosion	1 (1.3%)
Large bladder capacity with no sensation	1 (1.3%)
Diverticulum	1 (1.3%)
Small bladder capacity	1 (1.3%)
Multi-fibroid uterus bulging into bladder	1 (1.3%)
Pain on bladder filling	1 (1.3%)
Hunner’s lesions	1 (1.3%)

There were no immediate post-procedure complications. Nursing staff contacted patients within the first 24 hour over phone, and patients were also encouraged to report any issues through a direct email or phone, with all communications documented in patient charts. We reviewed charts 6 weeks post-procedure to look for any hospital presentations with complications, during which time no complications were reported. These findings demonstrate the feasibility and success of outpatient cystoscopy.

## Discussion

Cystoscopic evaluation of the lower urinary tract is an important part of investigation in women with urinary symptoms [7].

This retrospective review of 77 women who underwent outpatient flexible cystoscopy between May 2023 and October 2024 highlights the safety, feasibility and diagnostic utility of this service in a tertiary urogynaecology setting. The mean age of the patients was 52.5 years, ranging from 20 to 85 years, reflecting the broad spectrum of patients seen in clinical practice. Of note, 26% of our patients were above the age of 65 (ASA Class III or over) [8], who often present with multiple comorbidities that increase the risks associated with GA. Patient selection for an outpatient procedure versus under GA is important [9, 10]. Avoiding GA, not only reduces the risk of nosocomial infections, but it also encourages earlier mobilization and allows patients the benefit of recovering at home, should they want to [10, 11]. Additionally, it is a cost-effective measure [9, 10]. Furthermore, some evidence states that in the older population, there may be an associated risk of increase in rate of global cognitive decline following a GA [12]. Therefore, by performing the procedure in an outpatient setting, this service accommodates this vulnerable cohort, ensuring their safety while delivering essential diagnostic care.

Importantly, all procedures were successfully completed. There were no abandoned procedures secondary to patient discomfort. This shows appropriate patient selection for this outpatient procedure, therefore underscoring the patient-centred approach and efficacy of this service.

Table 1 highlights the indications for referrals, emphasizing the role of flexible cystoscopy in evaluating a range of lower urinary tract symptoms and suspected bladder pathologies.

Cystoscopy findings demonstrated that 100% of patients had a normal urothelium (non-cancerous pathology), suggesting that a significant proportion of women were reassured following this diagnostic evaluation. Among those with abnormal findings, trabeculations were the most frequent abnormality (39%) which are often associated with bladder outlet obstruction or long-standing BPS or interstitial cystitis [13]. Glomerulations were identified in 9% of patients, typically indicative of BPS or interstitial cystitis [14], while other findings included hyperaemia (8%) and cystitis cystica (3%). Rare findings such as diverticulum, Hunner’s lesions and mesh erosion were also identified, highlighting the diagnostic range of flexible cystoscopy in detecting both common and uncommon pathologies.

The quoted risks for this procedure include pain (1 in 10), bleeding (1 in 10), UTIs needing antibiotics (up to 1 in 50), bladder perforation (less than 1 in 50) or temporary catheterisation (less than 1 in 50) [1, 6]. We followed our patients over a period of 6 weeks. Notably, there were no post-procedure complications, affirming the safety of performing flexible cystoscopy in an outpatient setting. The absence of adverse events also highlights the adherence of the unit to international guidelines, including those outlined by the British Association of Urological Nurses (BAUN) and the British Association of Urological Surgeons (BAUS), particularly in terms of patient selection, technique and post-procedure care [6].

We also noted that in the first year after introduction of this service,36 patients underwent flexible cystoscopy compared to 41 patients in the following six months. This shows over two-fold (113%) increases in the workload highlighting the increasing need for this service.

We performed a cost–benefit analysis. In our hospital, the approximate cost of inpatient cystoscopy is € 1644 per patient compared to € 433 as an outpatient. This allowed for a saving of approximately € 1211 per patient, leading to a saving of over € 93,000 in a period of 18 months (Appendix 2). Our unit utilizes single use flexible cystoscope. In 2021, Zhuo et al. had a similar study showing cost-effectiveness of single use flexible cystoscopes over reusable ones [15].

These results reinforce the feasibility and value of outpatient flexible cystoscopy as a diagnostic tool in a tertiary urogynaecology setting. The high completion rate, absence of complications and the ability to provide reassurance or identify actionable findings highlights its importance in the efficient management of urogynaecology conditions. We acknowledge that our study involves a relatively small cohort of 77 patients; however, the substantial increase in service utilization over time reflects the growing demand for this diagnostic approach. Future work could focus on patient reported outcomes, such as satisfaction and symptom resolution, to further enhance the quality of care provided.

## Conclusion

Cystoscopy is a critical skill for gynaecologists, particularly those specializing in urogynaecology. Offering this service in an outpatient setting provides significant advantages for both patients and the hospital. Outpatient cystoscopy is tolerable and eliminates the need for inpatient admission. Additionally, it reduces costs by obviating the requirement for an anaesthetic team and inpatient admission. Patients also avoid the risks associated with GA, making it a safer alternative.

As demonstrated by our results, outpatient cystoscopy is a low-risk and safe diagnostic procedure, with no post-procedure complications observed in this study. Furthermore, it provides valuable diagnostic information for urogynaecologists, enabling the formulation of timely and effective management plans tailored to the patient’s condition.

## Appendix 2

Table 3

**Table 3:** Cost-benefit analysis

	Inpatient costs	Outpatient costs	Total savings
Single patient	€ 1644	€ 433	€ 1211
All patients (77 patients)	€ 126,588	€ 33,341	€ 93,247
